# Supplementary material for: Experimental vaccination by single dose sporozoite injection of blood-stage attenuated malaria parasites
Source: EMBO Mol Med. 2024 Aug 5;16(9):2060–79. doi: 10.1038/s44321-024-00101-6 (PMC11392930; doi:10.1038/s44321-024-00101-6)
Supplement: Supplementary file 1 — Table EV1 [file 44321_2024_101_MOESM1_ESM.docx]

|  |  | 100 iRBC SWISS | | | | | | 100 iRBC C57BL/6 | | | |
| --- | --- | --- | --- | --- | --- | --- | --- | --- | --- | --- | --- |
|  |  | dpi reaching peak parasitemia clearing  (earliest-mean-latest) | | blood stage cleared  (earliest-mean-latest d) | | | dpi reaching peak parasitemia clearing  (earliest-mean-latest) | | | blood stage cleared  (earliest-mean-latest d) | |
| *trx2(-)* |  | n.a. | | n.a. | | | n.a. | | | n.a. | |
| *plasmepsin IV(-)* |  | 16 | | 25 | | | n.a. | | | n.a. | |
| *atg 23(-)* |  | 17-20-20 | | 22-23-23 | | | n.a. | | | n.a. | |
| *profilin(-)* |  | n.a. | | n.a. | | | 14-15-16 | | | 22-23-23 | |
| *bclp 1(-)* |  | 17-19-23 | | 35-37-39 | | | n.a. | | | n.a. | |
| *man-1-P-GT(-)* |  | n.a. | | n.a. | | | 21-23-26 | | | 32-32-33 | |
| *U2 snRNP(-)* |  | 15-20-22 | | 22-26-30 | | | 16-19-22 | | | 20-25-30 | |
| *dgk(-)* |  | 16-18-21 | | 22-27-31 | | | 14-19-30 | | | 24-28-36 | |
| *hgprt(-)* |  | 15-17-19 | | 20-23-24 | | | 14-15-15 | | | 17 | |
| *v-type PPase(-)* |  | 13 | | 21 | | | n.a. | | | n.a. | |
| *app(-)* |  | 16-18-20 | | 20-21-23 | | | 14-14-15 | | | 18-21-23 | |
| *lap(-)* |  | 17-18-19 | | 20-21-23 | | | 14-15-15 | | | 19-20-20 | |
|  | Natural transmission  C57BL/6 | | | | 1,000 salivary gland sporozoites i.v.  C57BL/6 | | | | 10,000 salivary gland sporozoites i.v.  SWISS | | |
|  | dpi reaching peak parasitemia clearing  (earliest-mean-latest) | | blood stage cleared  (earliest-mean-latest d) | | dpi reaching peak parasitemia clearing  (earliest-mean-latest) | blood stage cleared  (earliest-mean-latest d) | | | dpi reaching peak parasitemia clearing  (earliest-mean-latest) | | blood stage cleared  (earliest-mean-latest d) |
| *app(-)* | 12-14-18 | | 20-22-24 | | 13-17-31 | 17-27-46 | | | 12-16-19 | | 17-23-38 |
| *lap(-)* | 9-15-25 | | 17-22-32 | | 12-17-18 | 20-23-27 | | | 15-16-18 | | 17-20-23 |
