# Supplementary material for: Experimental vaccination by single dose sporozoite injection of blood-stage attenuated malaria parasites
Source: EMBO Mol Med. 2024 Aug 5;16(9):2060–79. doi: 10.1038/s44321-024-00101-6 (PMC11392930; doi:10.1038/s44321-024-00101-6)
Supplement: Supplementary file 2 — Table EV2 [file 44321_2024_101_MOESM2_ESM.docx]

| Parasite line | Mouse strain | Immunized through | Experiment | Animals clearing/  animals infected | Total number of animals clearing/ animals infected |
| --- | --- | --- | --- | --- | --- |
| *app(-)* | SWISS | 100 iRBC | Appendix Fig. S6 | 3/4 | 50/52 |
|  |  | 10,000 sporozoites | Appendix Fig. S9 | 6/6 |  |
|  | C57BL/6 | 100 iRBC | Fig. 2 A | 4/4 |  |
|  |  | Natural transmission | Fig. 2 D,M | 13/14 |  |
|  |  | 1,000 sporozoites | Fig. 2 G | 24/24 |  |
| *lap(-)* | SWISS | 100 iRBC | Appendix Fig. S6 | 4/4 | 84/84 |
|  |  | 10,000 sporozoites | Appendix Fig. S9 | 4/4 |  |
|  | C57BL/6 | 100 iRBC | Fig. 2 A | 4/4 |  |
|  |  |  | Fig. 4 B | 4/4 |  |
|  |  | 1,000 iRBC | Fig. EV3 | 4/4 |  |
|  |  | Natural transmission | Fig. 2 D,M | 16/16 |  |
|  |  | 1,000 sporozoites | Fig. 2 G | 24/24 |  |
|  |  |  | Fig. 4 G,H | 20/20 |  |
|  |  | 10,000 sporozoites | Fig. EV3 | 4/4 |  |
