# Supplementary material for: Experimental vaccination by single dose sporozoite injection of blood-stage attenuated malaria parasites
Source: EMBO Mol Med. 2024 Aug 5;16(9):2060–79. doi: 10.1038/s44321-024-00101-6 (PMC11392930; doi:10.1038/s44321-024-00101-6)
Supplement: Supplementary file 3 — Appendix [file 44321_2024_101_MOESM3_ESM.pdf]

Table of contents

**APPENDIX FIG. S1.**	**3**
**APPENDIX FIG. S2.**	**4**
**APPENDIX FIG. S3.**	**5**
**APPENDIX FIG. S4.**	**7**
**APPENDIX FIG. S5.**	**8**
**APPENDIX FIG. S6.**	**9**
**APPENDIX FIG. S7.**	**10**
**APPENDIX FIG. S8**	**11**
**APPENDIX FIG. S9.**	**12**
**APPENDIX FIG. S10**	**13**
**APPENDIX FIG. S11.**	**14**
**APPENDIX FIG. S12**	**16**
**APPENDIX FIG. S13.**	**17**
**APPENDIX TABLE S1.**	**18**
**APPENDIX TABLE S2.**	**22**
**APPENDIX TABLE S3.**	**23**
**APPENDIX TABLE S4.**	**30**
**APPENDIX TABLE S5.**	**31**
**APPENDIX TABLE S6.**	**32**
**APPENDIX TABLE S7.**	**33**
**APPENDIX TABLE S8**	**34**

APPENDIX TABLE S9. 35

APPENDIX TABLE S10. 36

Appendix Fig. S1.

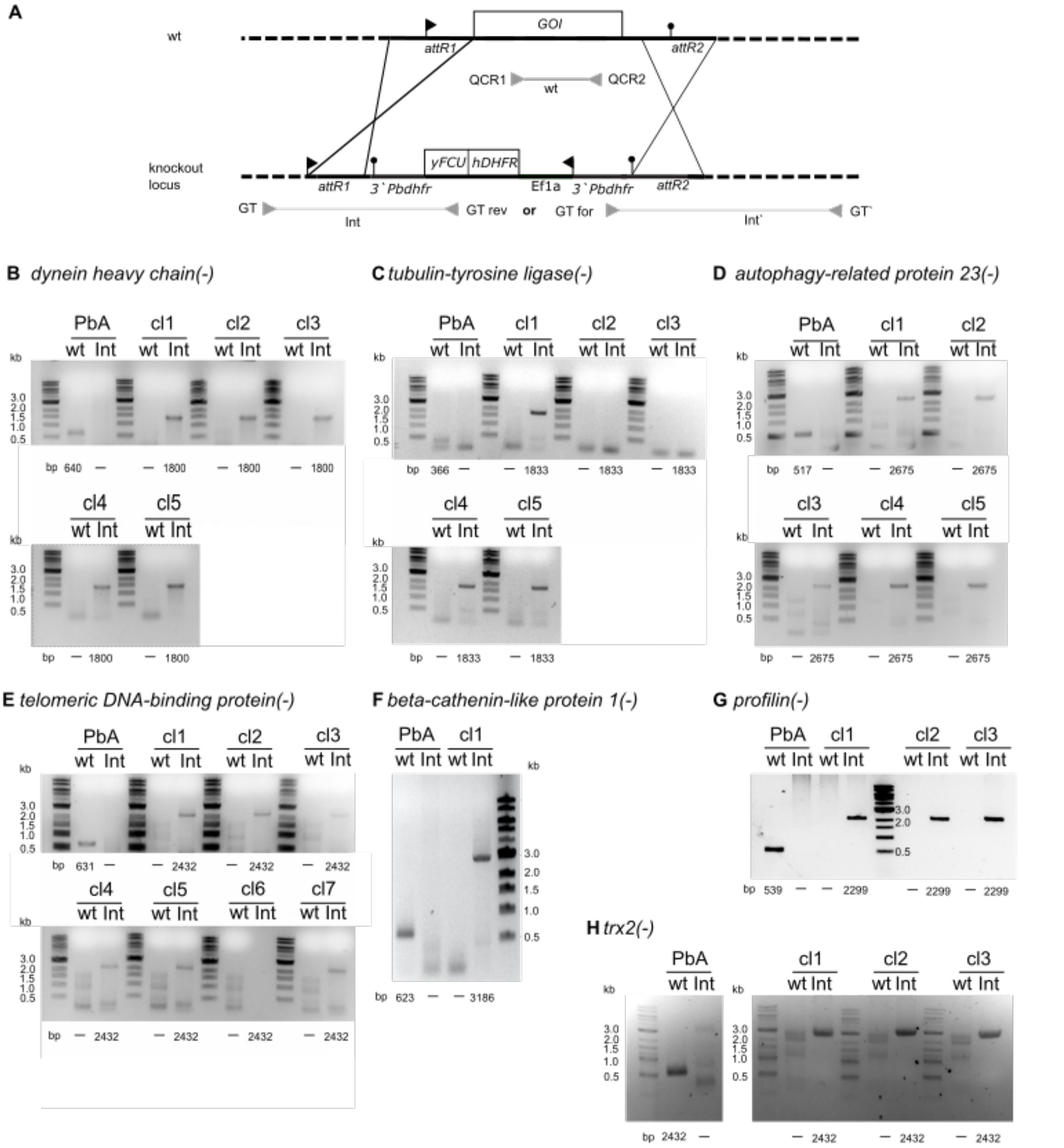

Appendix Fig. S1. Generation of *P. berghei* mutants. (A) Schematic showing chromosome locus of wild type (top) and mutant after integration of a PlasmoGEM vector (bottom). Primers used for PCR and expected amplicons are indicated. **(B-G)** Genotyping PCRs verify correct integration for indicated clonal gene-deletion parasite lines. Expected amplicon sizes below the gels; for primer combinations see Appendix Table S3. Abbreviations: GOI gene of interest; attR attachment sites for recombination; QCR quality control R; GT genotyping; rev reverse; int integration; Pbdhfr *Plasmodium berghei* dihydrofolate reductase; yFCU yeast cytosine deaminase and uridyl phosphoribosyl transferase; hDHFR human dihydrofolate reductase; cl clone; bp base pairs; kb kilo base pairs.

Appendix Fig. S2.

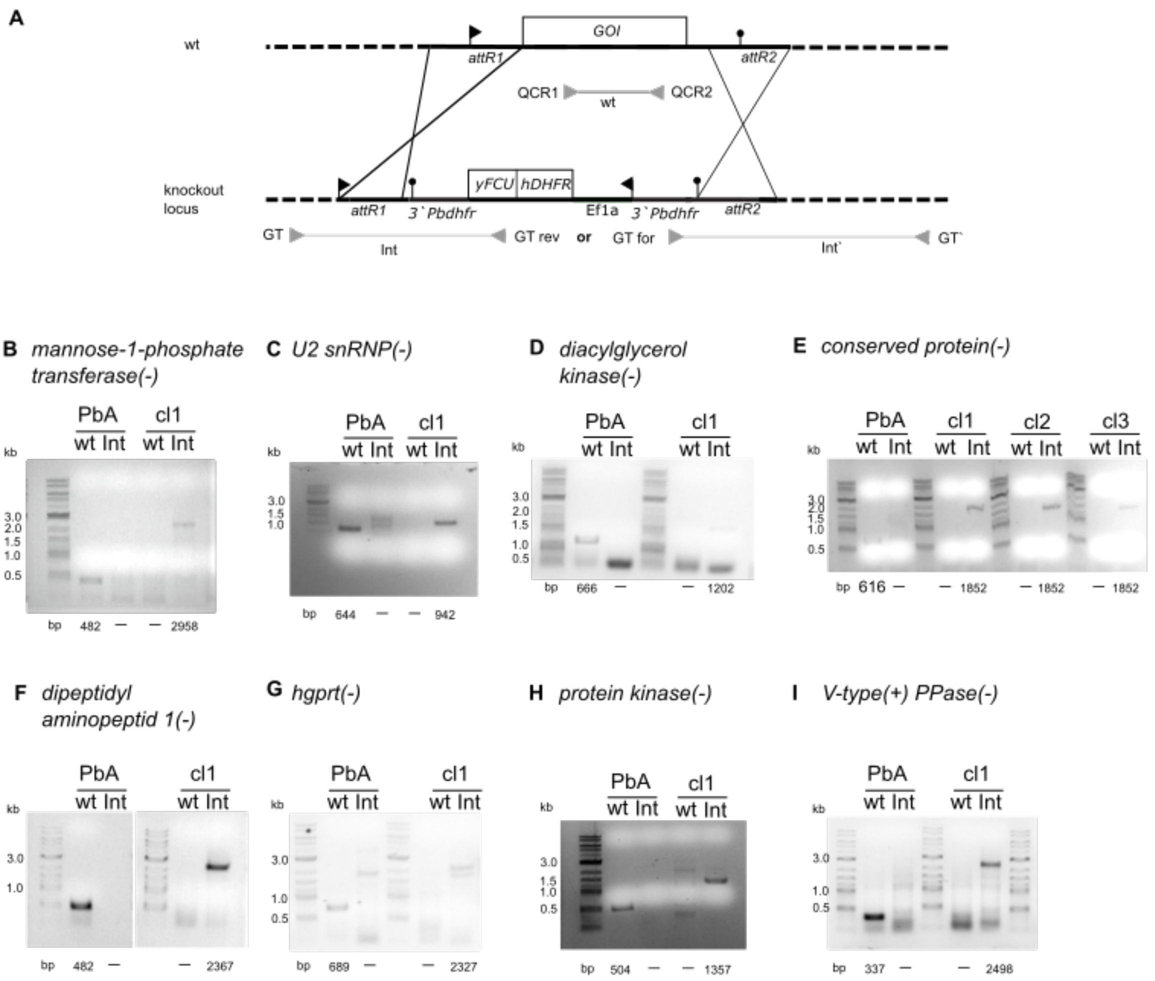

Appendix Fig. S2. Generation of *P. berghei* mutants. (A) Schematic showing chromosome locus of wild type (top) and mutant after integration of a PlasmoGEM vector (bottom). Primers used for PCR and expected amplicons are indicated. **(B-G)** Genotyping PCRs verify correct integration for indicated clonal gene-deletion parasite lines. Expected amplicon sizes below the gels; for primer combinations see Appendix Table S3. Abbreviations: GOI gene of interest; attR attachment sites for recombination; QCR quality control R; GT genotyping; rev reverse; int integration; Pbdhfr *Plasmodium berghei* dihydrofolate reductase; yFCU yeast cytosine deaminase and uridyl phosphoribosyl transferase; hDHFR human dihydrofolate reductase; cl clone; bp base pairs; kb kilo base pairs.

Appendix Fig. S3.

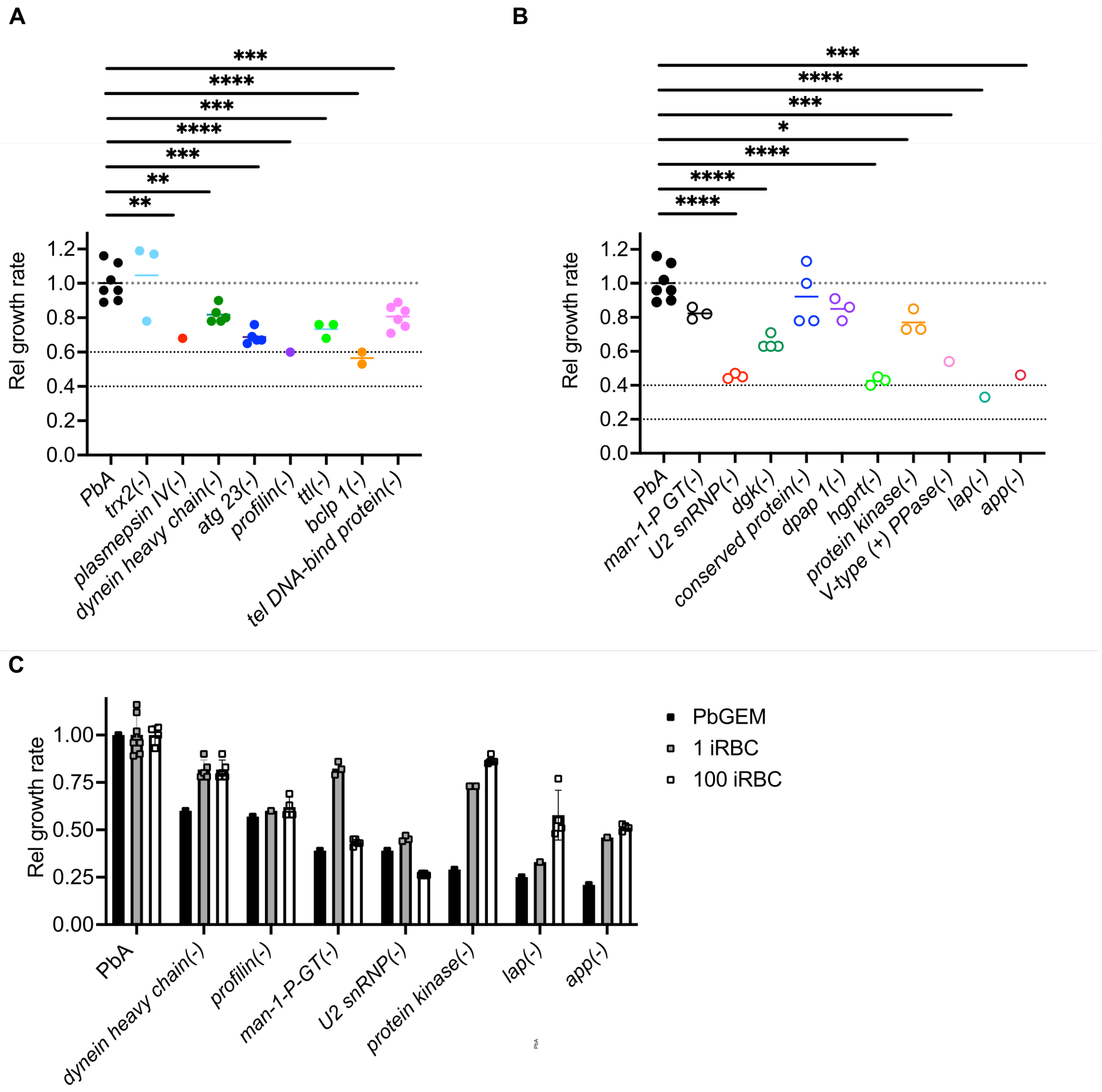

Appendix Figure S3. Relative growth rates of gene-deletion mutants are reduced compared to PbA parasites. (A) Intraerythrocytic growth of clonal gene-deletion mutants with a reported PlasmoGEM growth rate of 0.4-0.8. The clonal growth rate was between 0.9 and 0.5. The growth rate of *plasmepsin IV(-)* was added according to (Spaccapelo *et al*, 2010). One data point represents one infection with one iRBC per mouse. Ten SWISS mice were initially infected but only 3/10 *trx2(-)*, 5/10 *dynein heavy chain(-)*, 5/10 *atg 23(-)*, 1/10 *profilin(-)*, 3/10 *ttl(-)*, 2/10 *bclp 1(-)* and 6/10 *tel DNA-bind protein(-)* infected mice became blood stage positive. Relative growth rates were compared to PbA by One-way ANOVA. **(B)** Intraerythrocytic growth of clonal gene-deletion mutants with a reported PlasmoGEM growth rate of 0.2-0.4. The clonal growth rate was between 1.1 and 0.4. The growth rates of *lap(-)* and *app(-)* was added according to (Lin *et al*, 2015). One data point represents one infection with one iRBC

per mouse. Ten SWISS were initially infected but not all mice became blood stage positive. 3/10 *man-1-P GT(-)*, 3/10 *U2 snRNP(-)*, 5/10 *dgk(-)*, 3/10 *conserved protein(-)* and *1/10 V-type(+) PPase(-)* infected mice became blood stage positive. Relative growth rates were compared to PbA by One-way ANOVA. PbA data are the same as in (A) **(C)** Comparison of relative growth rates from (Bushell *et al*, 2017) and our data from infections with 1 iRBC (Figure S3 A, B) or 100 iRBC (C57BL/6) (Fig. 2, Appendix Fig. S7, Appendix Table S5). The relative growth rate was calculated by normalization to wild type growth rates obtained from similar infections.

Appendix Fig. S4.

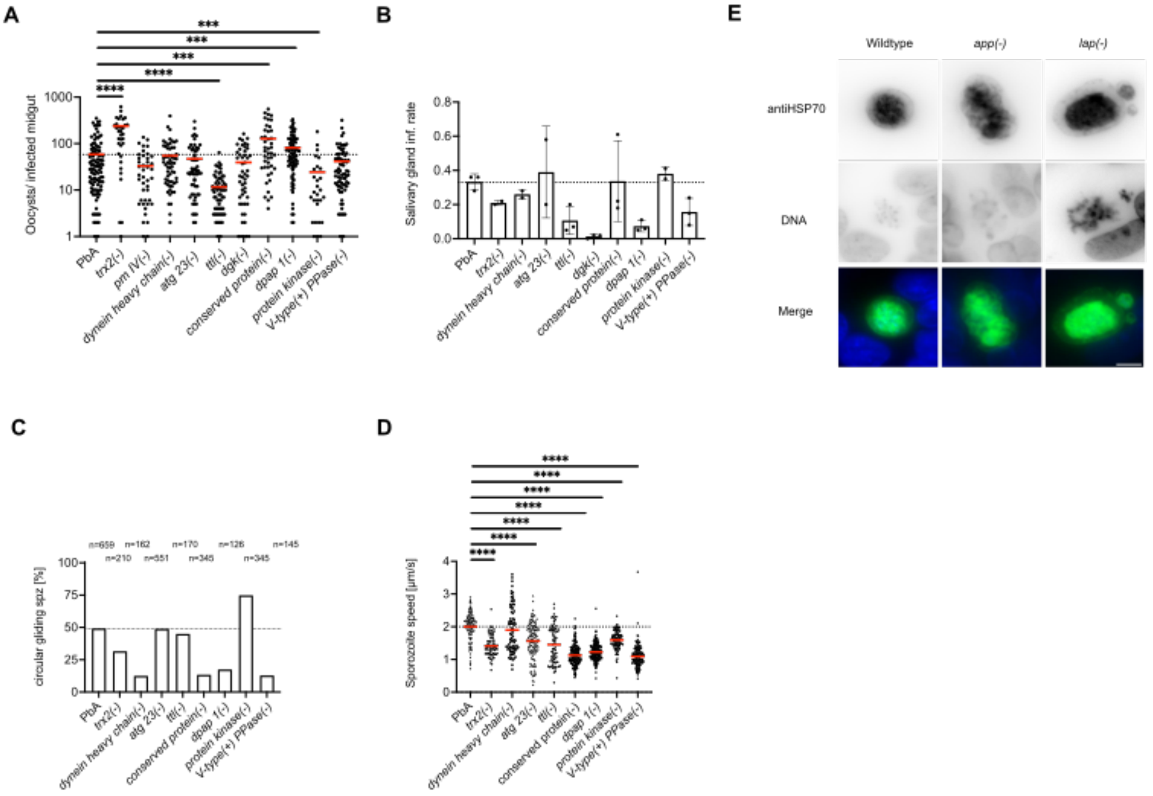

Appendix Fig. S4. Characterization of mutant parasite development through mosquito and liver stages. (A) Oocyst numbers per infected mosquito. Dotted line depicts mean oocyst numbers of wild type parasites. Two-tailored Mann-Whitney test.***,p<0.001, ****, p<0.0001. **(B)** Salivary gland infection rate. *trx 2(-)*, *dynein heavy chain(-)*, *ttl(-)*, *dgk(-)*, *dpap 1(-)* and *V-type(+) PPase(-)* sporozoites display a reduced capacity to invade salivary glands. **(C)** Motility of salivary gland-derived sporozoites. A reduced number of *dynein heavy chain(-)*, *conserved protein(-)*, *dpap 1(-)* and *V-type(+) PPase(-)* sporozoites are able to productively move in a 2D environment although they reach similar speed compared to wild type and other gene-deletion parasite lines **(D)**. Dotted line indicates mean of wild type sporozoite speed. Two-tailored Mann-Whitney test, ****, p<0.0001. **(E)** Representative images of *in vitro* developing wild type, *app(-)* and *lap(-)* liver stage parasites. Immunofluorescence assay 48h post-invasion of HepG2 cells. Nuclei were stained using Hoechst, liver stage cytoplasm using a parasite specific anti-*Pb*HSP70 antibody. Scale bar 10 µm.

Appendix Fig. S5.

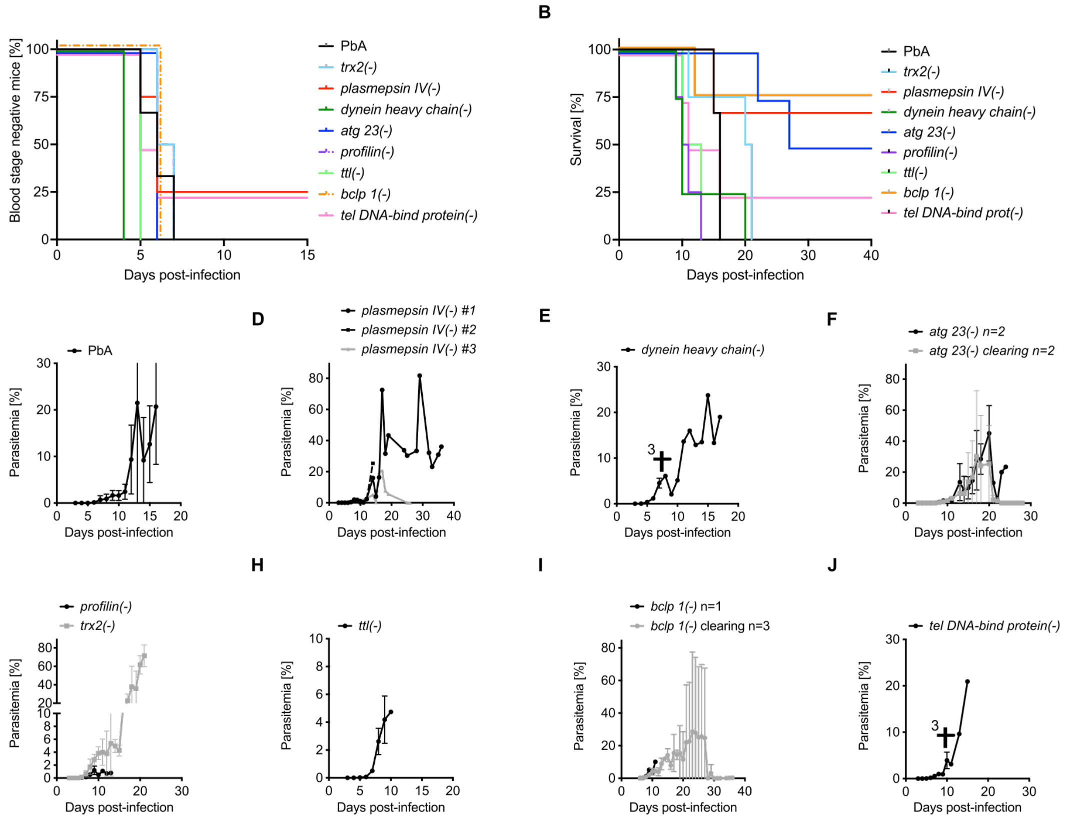

Appendix Fig. S5. Prepatency, parasitemia curves and survival of SWISS mice infected with 100 intraerythrocytic gene-deletion mutants with an expected growth rate of 0.4-0.6. (A) Percentage of blood stage negative SWISSinfected with 100 iRBC of PbA, *plasmepsin IV(-)*, *trx2(-)*, *dynein heavy chain(-)*, *atg 23(-)*, *profilin(-)*, *ttl(-)bclp 1(-)* or *tel DNA-bind protein(-).* Four to six days post infection all mice (n=4) intravenously infected with 100 iRBC of PbA, *trx2(-)*, *dynein heavy chain(-)*, *atg 23(-)*, *profilin(-)*, *ttl(-)*, *bclp 1(-)* became blood stage positive. 3 out of 4 mice became blood stage positive after infection with 100 iRBC of *plasmepsin IV(-)* or *tel DNA-bind protein(-)*. **(B)** Survival of mice infected in (A). All mice infected with 100 iRBC of PbA, *dynein heavy chain(-)*, *profilin(-)*, *trx2(-)*, *ttl(-)* or *tel DNA-bind protein(-)* died between day 9 and day 21 post infection. In contrast, some mice survived and cleared the infection caused by *plasmespin IV(-), atg 23(-)* or *bclp 1(-)* parasites. **(C-J)** Course of infection of SWISS mice infected in (A). Parasitemia is shown either as mean parasitemia of all mice dying (black) or clearing (grey) except (D) where individual parasitemia curves of all mice infected are displayed. For better visibility data were nudged to prevent overlap in (A) and (B).

Appendix Fig. S6.

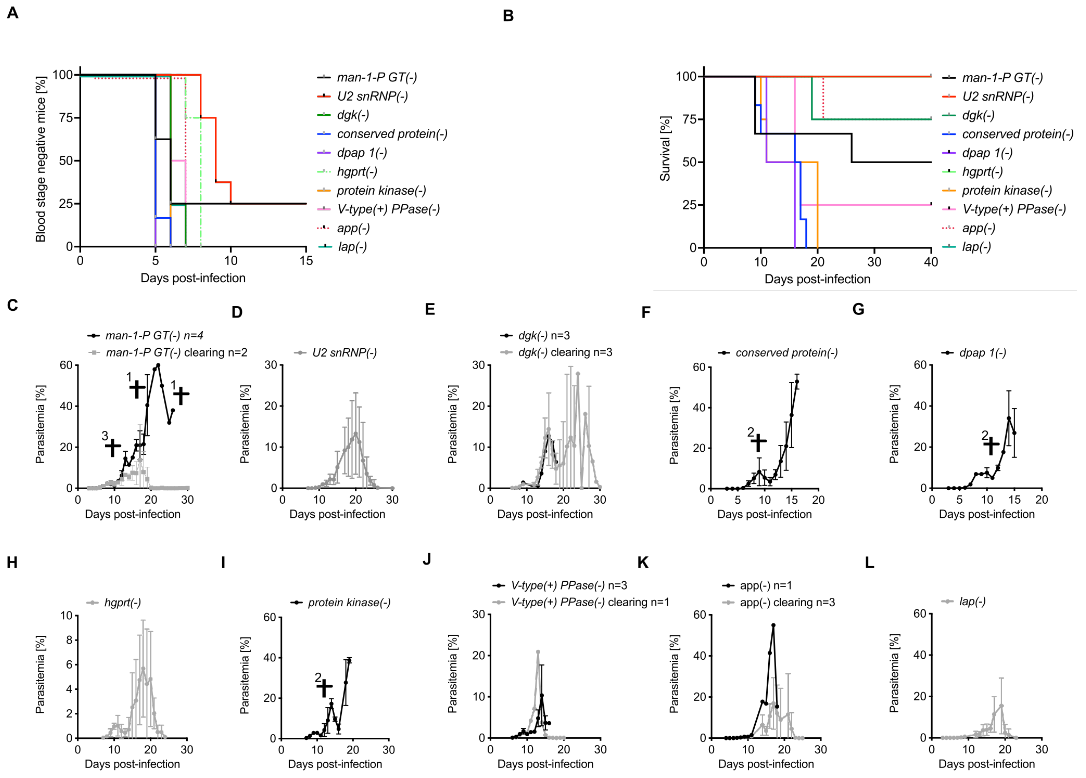

Appendix Fig. S6. Prepatency, survival and course of infection in SWISS mice infected with 100 intraerythrocytic gene-deletion mutants (expected growth rate 0.2-0.4). (A) Percentage of blood stage negative SWISS infected with 100 iRBC of *dgk(-)*, *dpap 1(-), hgprt(-), protein kinase(-), V-type(+) PPase(-), lap(-)*, *app(-)*, *conserved protein(-)*, *man-1-P GT(-)* or *U2 snRNP(-)*. Five to eight days post-infection all mice (n=4) infected with 100 iRBC of *dgk(-)*, *dpap 1(-), hgprt(-), protein kinase(-), V-type(+) PPase(-), lap(-)* or *app(-)* became blood stage positive as well as all (n=6) mice infected with 100 iRBC of *conserved protein(-).* Six out of eight mice infected with 100 iRBC of *man-1-P GT(-)* or *U2 snRNP(-)* became blood stage positive between day five and ten post-infection. **(B)** Survival of mice infected in (A). All mice infected with 100 iRBC of *conserved protein(-)*, *dpap 1(-)* or *protein kinase(-)* died between day 9 and day 20 post infection. In contrast some mice survived and cleared the infection caused by *man-1-P-GT(-), dgk(-)*, *V-type(+) PPase(-)* or *app(-)* parasites. All mice infected with *hgprt(-), U2 snRNP(-)* or *lap(-)* survived and cleared the infection. **(C-L)** Course of infection of SWISS mice infected in (A). Parasitemia is shown as mean parasitemia of all mice dying (black) or clearing (grey). For better visibility data were nudged to prevent overlap in (A).

Appendix Fig. S7.

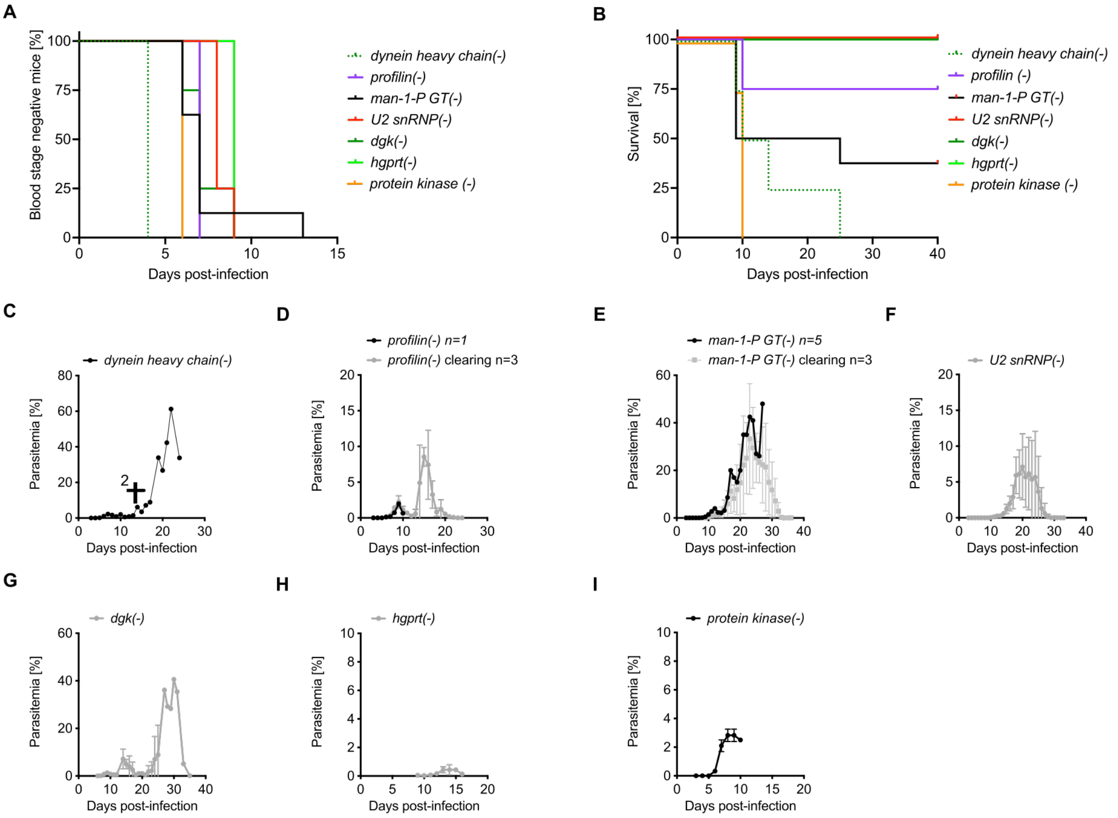

Appendix Fig. S7. Prepatency and survival of C57BL/6 mice infected with 100 intraerythrocytic gene-deletion mutants and the corresponding parasitemia curves. (A) Percentage of blood stage negative C57BL/6 mice infected intravenously with 100 iRBC. Four to thirteen days post infection all mice (n=4 for *dynein heavy chain(-)*, *profilin(-)*, *man-1-P GT(-)*, *dgk(-)* and *protein kinase(-)*; n=8 for *man-1-P-GT(-)* and *U2 snRNP(-))* became blood stage positive. 3 of 4 C57BL/6 mice infected with 100 iRBC of *hpgrt(-)* became blood stage positive. **(B)** Survival of mice infected in (A). All C57BL/6 mice infected with 100 iRBC of *dynein heavy chain(-)* and *protein kinase(-)* died between day 10 and day 25 post infection. In contrast some mice survived and cleared the infection caused by *profilin(-)* or *man-1-P GT(-)* parasites. All mice infected with 100 iRBC of *U2 snRNP(-)*, *dgk(-)* or *hpgrt(-)* were able to survive and became blood stage negative, latest by day 35. **(C-I)** Course of infection of C57BL/6 mice infected in (A). Parasitemia is shown either as mean parasitemia of all mice dying (black) or clearing (clearing). For better visibility data were nudged to prevent overlap (B).

Appendix Fig. S8

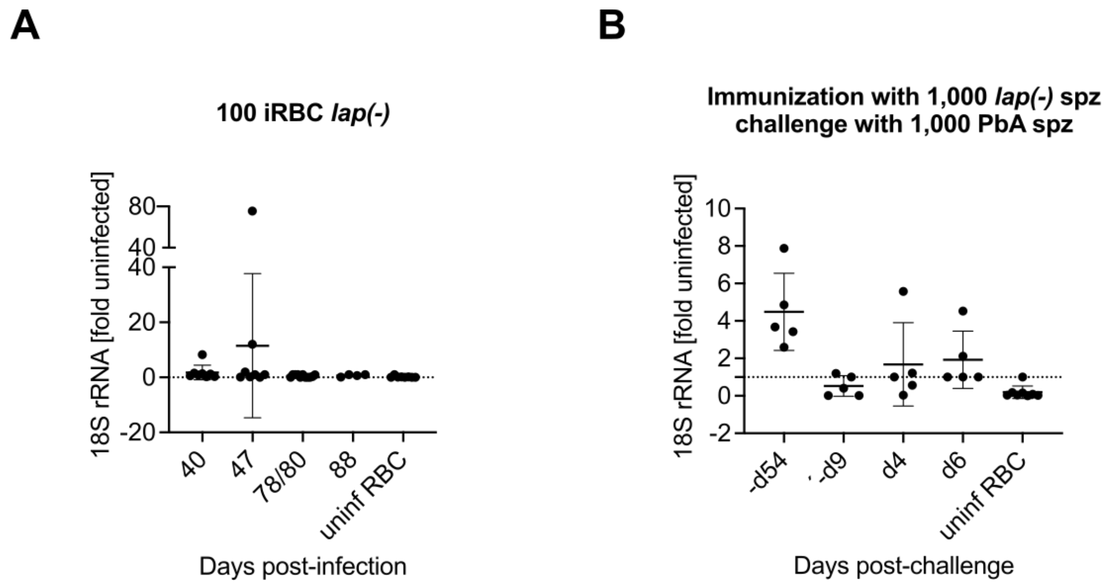

Appendix Fig. S8. qPCR on blood lysates of previously infected or challenged mice. (A) qPCR on mice infected with 100 *lap(-)* iRBC *i.v.*. Although mice were blood stage negative by Giemsa-stained blood smears d21 post-infection a sub-patent infection was readily detectable by qPCR in some mice tested at d40 post-infection. At d47 post-infection we were not able to detect parasites in 6/8 mice tested. From d78 post-infection onwards all mice were negative by qPCR. **(B)** qPCR data on *lap(-)* immunized mice during immunization (-d54), before (-d9) and after (d4 and d6) challenge with with 1,000 wild type sporozoites. Importantly all mice were blood stage negative by qPCR nine days (-d9) before challenge. On d4 post-challenge 1/4 immunized mice was blood stage positive. On d6 post-challenge 2/4 mice were blood stage positive detected by qPCR as well as by Giemsa-stained blood smear. Individual dots represent individual mice. Ct values of uninfected RBC from naïve mice had a mean of 33 ± 2. Ct values of infected animals were normalized to the highest ct value (30) of naïve mice, depicted as dotted line.

Appendix Fig. S9.

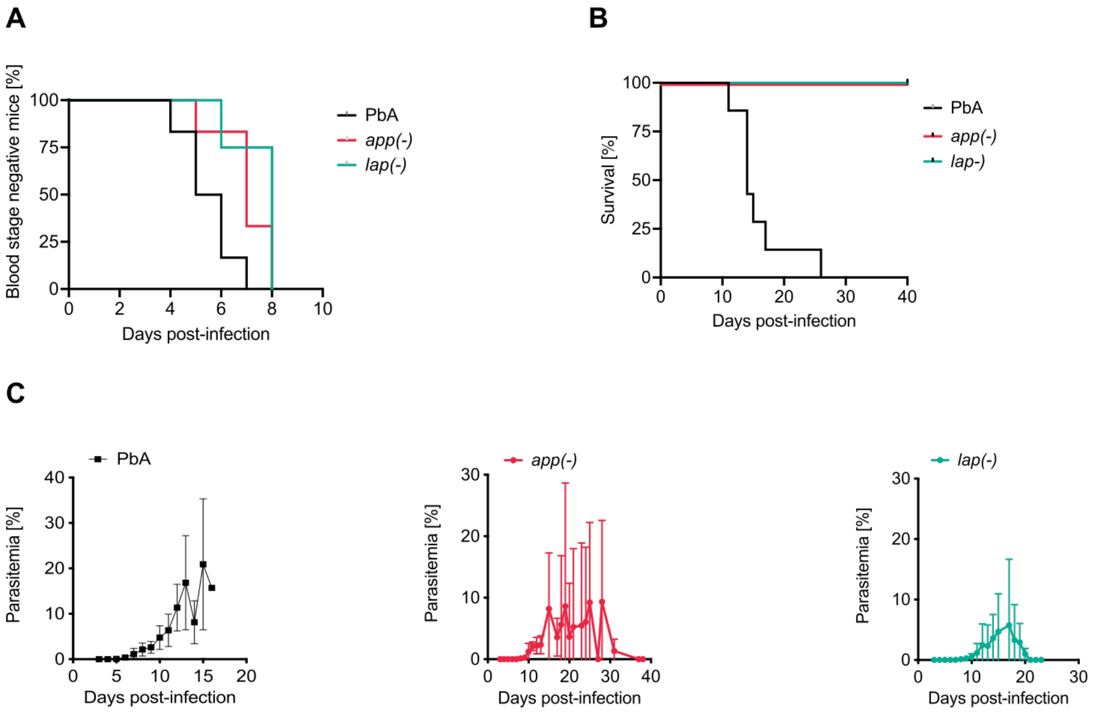

Appendix Fig. S9. Prepatency and survival of SWISS mice infected by intravenous injection of 10,000 salivary gland derived sporozoites and the corresponding parasitemia curves. (A) Percentage of blood stage negative SWISS mice (n=4) infected with 10,000 PbA, *lap(-)* or *app(-)* sporozoites *i.v.*. Mice became blood stage positive between day four and eight post infection. **(B)** Survival of SWISS mice infected in (A). All mice infected with PbA died between day nine and twenty-seven. In contrast, infections with 10,000 *lap(-)* or *app(-)* sporozoites were cleared and mice became blood stage negative between d25 and d49 post-infection. **(C)** Parasitemia curves of infected mice shown as mean parasitemia of all mice infected ± SD. For better visibility data were nudged in B.

Appendix Fig. S10

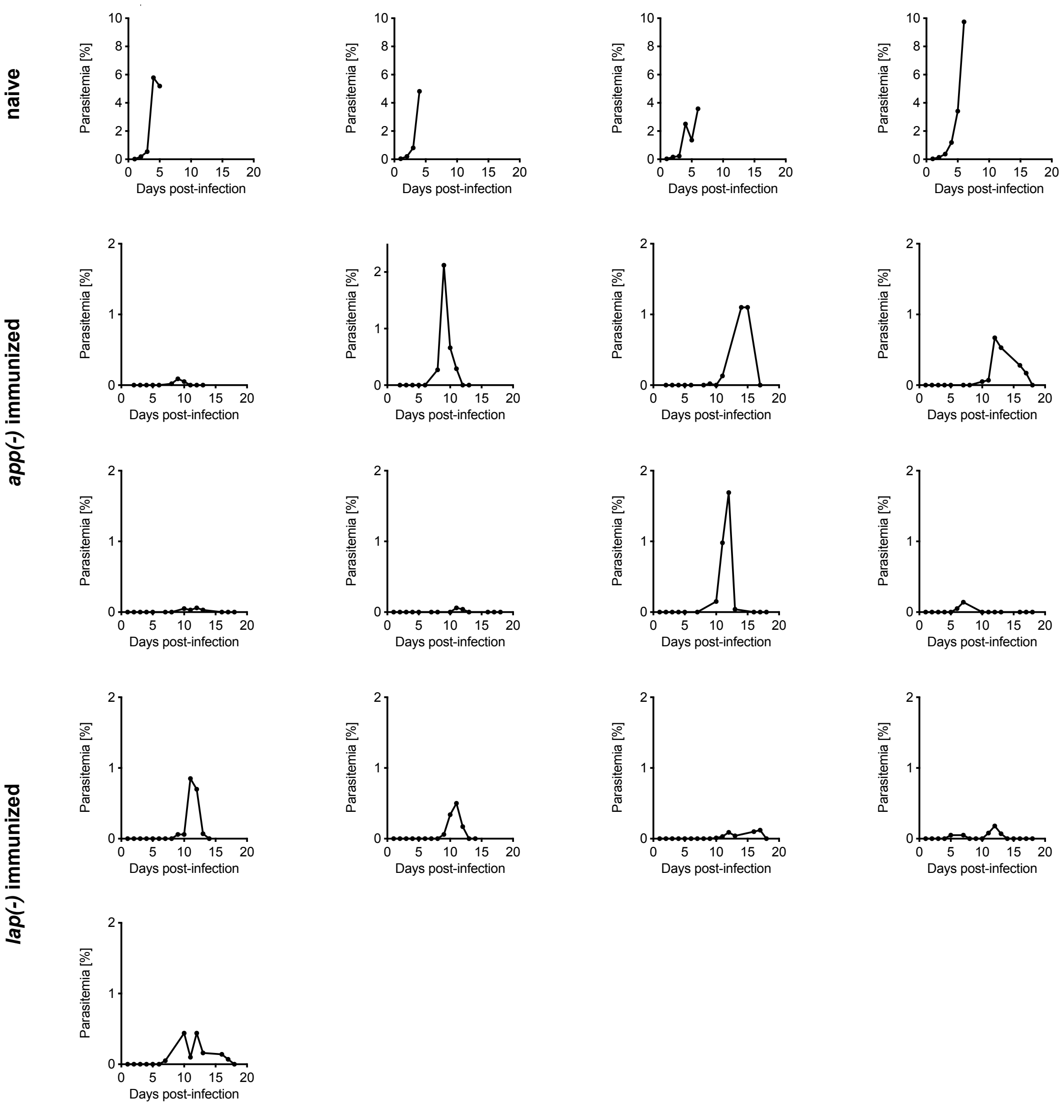

Appendix Fig. S10. Individual parasitemia curves of blood stage positive mice challenged 90 days post-infection.

Appendix Fig. S11.

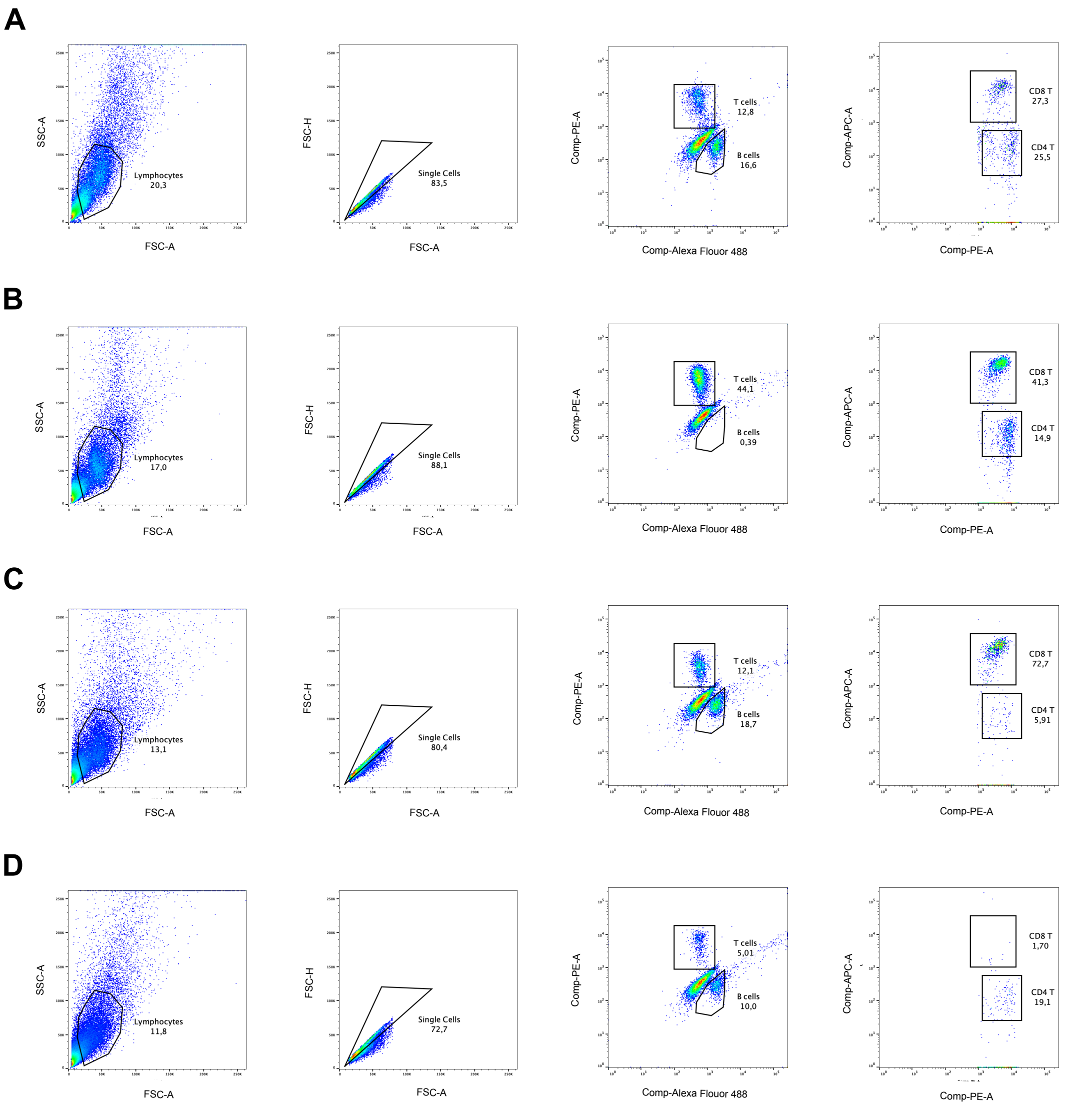

Appendix Fig. S11. Gating strategy showing the depletion of B cells, CD4⁺ and CD8⁺ T cells in mice. (A) Representative flow cytometry plots of a naïve mouse. Cells were gated first on lymphocytes followed by elimination of doublets by gating on FSC-A and FSC-H. B cells were identified by staining with anti-mouse CD19-Alexa Fluor 488. T cells were identified by staining with CD3ε-PE followed by identification of $CD8^+$ T cells by staining with CD8a-APC. $CD4^+$ T cells were classified based on being CD3ε positive and CD8a negative. **(B)** Representative flow cytometry plots of a B-cell depleted mouse. Depletion of B-cells was confirmed by flow cytometry every seventh day**.** A representative flow cytometry plot is shown. **(C)** Representative flow cytometry plots of a $CD4^+$ T cell depleted mouse. Depletion of $CD4^+$ T cells was confirmed by flow cytometry every fifth day. A representative flow cytometry plot is shown. **(D)** Representative flow cytometry plots of a $CD8^+$ T cell depleted mouse. Depletion of $CD8^+$ T cells was

confirmed by flow cytometry every fifth day. A representative flow cytometry plot is shown.

Appendix Fig. S12

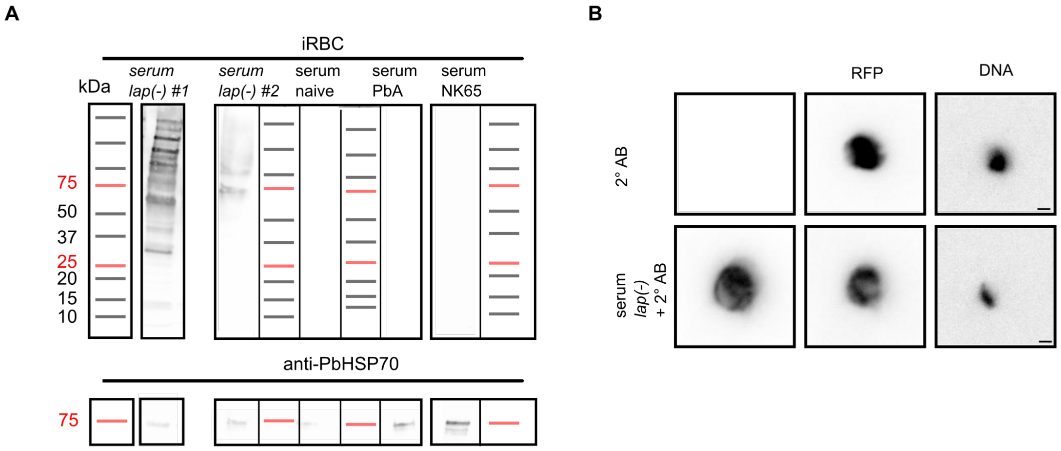

Appendix Fig. S12. Sera of immunized mice recognize parasite specific proteins. (A) Western blot on blood stage parasite lysates. In contrast to sera from PbA-infected (d8 post infection), NK65-infected (d24 post infection) or naive mice, sera of two *lap(-)* immunized mice (#1 and #2) detect parasite specific proteins. **(B)** Immunofluorescence assay on infected RBCs. Nuclei were stained using Hoechst, RFP expressing parasites were stained using serum from a *lap(-)*-immunized mouse and an anti-mouse Alexa Fluor 488 antibody. As a negative control permeabilized iRBCs were incubated with Hoechst und anti-mouse Alexa Fluor 488 antibody only. Scale bar 1 µM.

Appendix Fig. S13.

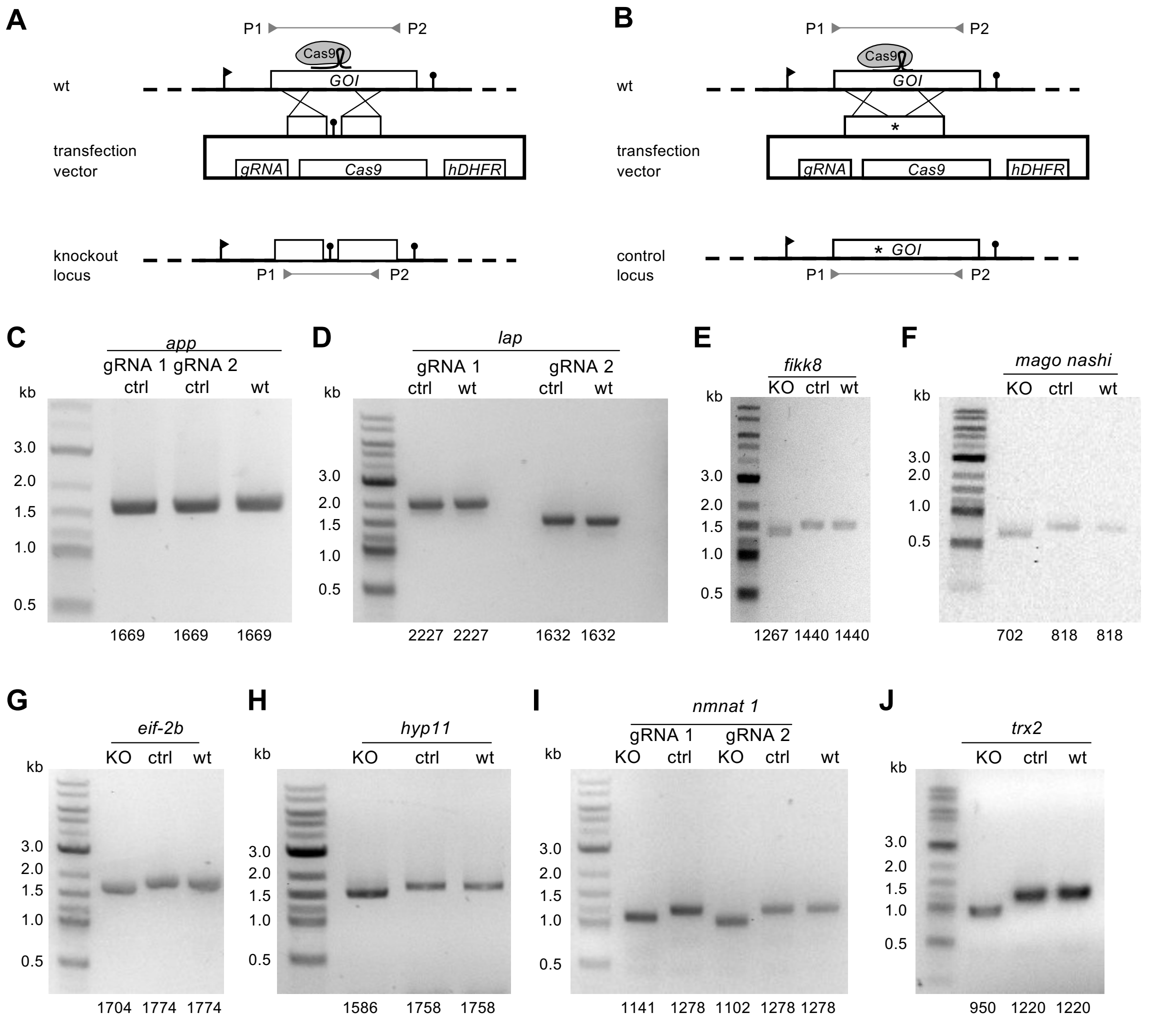

Appendix Fig. S13. Generation of slow-growing *P. falciparum* parasites. (A, B) Strategy for the generation of knockout (A) or control (B) parasite lines. Expression of Cas9 and gRNA introduces a cleavage in the gene of interest, which is repaired by (A) a repair template designed to introduce a deletion, a frameshift and a stop coding into the gene or (B) a repair template designed to only introduce a shielding mutation. Note that only the repair template itself integrates, not the plasmid containing foreign genetic material. The binding sites for genotyping primers P1 and P2 are indicated. GOI, gene of interest; hDHFR, human dihydrofolate reductase (resistance marker). **(C-J)** Genotyping PCRs verify the indicated knockout and control lines; expected amplicon sizes are indicated below the gels. Control PCR products were additionally sequenced to confirm introduced silent point mutations. Primer combinations: table S3. GOI, gene of interest; gRNA, guide RNA; hDHFR, human dihydrofolate reductase; KO, knockout; ctrl, control; wt, wild type.

Appendix Table S1.

	Gene of interest	PlasmoGEM Vector ID	Transfection attempts	Transfection succesfull	Limiting dilutions attempts
1.	*dynein heavy chain*	PbGEM-330283	2	yes	1
2.	*autophagy-related protein 23*	PbGEM-332739	1	yes	1
3.	*profilin*	PbGEM-286426	1	yes	1
4.	*tubulin tyrosine ligase*	PbGEM-287138	1	yes	1
5.	*telomeric DNA-binding protein*	PbGEM-304250	1	yes	1
6.	*beta-catenin like protein 1*	PbGEM-288226	1	yes	1
7.	*mannose-1-phosphate guanyltransferase*	PbGEM-037559	1	yes	1
8.	*diacylglycerol kinase*	PbGEM-265364	2	yes	1
9.	*U2 snRNP-associated SURP motif-containing protein*	PbGEM-243939	1	yes	1
10	*conserved protein*	PbGEM-533923	1	yes	1
11	*dipeptidyl aminopeptidase*	PbGEM-032467	1	yes	1
12	*hypoxanthine-guanine phosphoribosyl transferase*	PbGEM-336339	1	yes	1
13	*protein kinase*	PbGEM-259660	2	yes	1
14	*V-type (+)-translocating pyrophosphatase*	PbGEM-253763	3	yes	1
15	*thioredoxin 2*	PbGEM-317419	1	yes	1
16	*sec1 familiy protein*	PbGEM-232532	3	yes	5
17	*replication factor C subunit 2*	PbGEM-230189	3	yes	3
18	*alp2a*	PbGEM-264748	4	yes	1
19	*calmodulin-like protein*	PbGEM-330979	3	yes	4
20	*cs-domain protein*	PbGEM-331875	1	yes	1
21	*calcium-dependent protein kinase 5*	PbGEM-111682	4	no	n.a.

22	*phosphatidylinositol N-acetylglucosaminyltransferase*	PbGEM-090803	3	yes	1
23	*palmitoyltransferase DHHC4, putative*	PbGEM-065186	2	no	n.a.
24	*ras-related protein Rab-5B*	PbGEM-109737	2	no	n.a.
25	*mitochondrial carrier protein, putative*	PbGEM-254102	3	no	n.a.
26	*histone acetyltransferase*	PbGEM-242177	2	yes	2
27	*coatomer subunit gamma, putative*	PbGEM-260652	7	no	n.a.
28	*unknown*	PbGEM-268387	4	no	n.a.
29	*tRNA-YW synthesizing protein, putative*	PbGEM-261652	2	no	n.a.
30	*GPI-anchor transaminase*	PbGEM-240110	3	no	n.a.
31	*ww domain-binding protein 11, putative*	PbGEM-294664	6	yes	3
32	*conserved Plasmodium protein, unknown function*	PbGEM-335483	3	no	n.a.
33	*conserved Plasmodium protein, unknown function*	PbGEM-316571	2	yes	2
34	*multidrug resistance protein*	PbGEM-337155	2	no	n.a.
35	*cytoadherence linked asexual protein, putative*	PbGEM-256118	3	no	n.a.
36	*conserved Plasmodium protein, unknown function*	PbGEM-260988	3	no	n.a.
37	*sec24 subunit, putative*	PbGEM-246799	3	no	n.a.
38	*conserved Plasmodium protein, unknown function*	PbGEM-268387	1	no	n.a.
39	*conserved Plasmodium protein, unknown function*	PbGEM-298869	3	no	n.a.
40	*conserved Plasmodium protein, unknown function*	PbGEM-329659	3	no	n.a.
41	*M1-family alanyl aminopeptidase, putative*	PbGEM-109859	1	no	n.a.
42	*mago nashi protein homologue, putative*	PbGEM-235866	2	no	n.a.
43	*fbpA domain protein, putative*	PbGEM-323051	1	no	n.a.

44	*U6 snRNA phosphodiesterase, putative*	PbGEM-317307	1	no	n.a.
45	*protein kinase, putative*	PbGEM-337395	1	no	n.a.
46	*histone acetyltransferase subunit NuA4, putative*	PbGEM-321795	1	no	n.a.
47	*single-stranded DNA-binding protein, putative*	PbGEM-246443	1	no	n.a.
48	*bir protein;PIR protein*	PbGEM-537051	1	no	n.a.

Appendix Table S1. Overview of all performed transfections and limiting dilutions. Abbreviations: n.a., not applicable.

Appendix Table S2.

	Parasite line	Gene ID			Plasmo GEM ID
		P. berghei	*P. falciparum*	*P. knowlesi*	
1.	*trx2(-)*	PBANKA_ 1358000	PF3D7_ 1345100	PKNH_ 1256200	PbGEM -317419
2.	*plasmepsin IV(-)*	PBANKA_ 1034400	PF3D7_ 1408100	PKNH_ 1350300	n.a.
3.	*dynein heavy chain(-)*	PBANKA_ 0615700	PF3D7_ 0718000	PKNH_ 0214800	PbGEM- 330283
4.	*atg 23(-)*	PBANKA_ 0921700	PF3D7_ 1126700	PKNH_ 0924600	PbGEM- 332739
5.	*profilin(-)*	PBANKA_ 0833000	PF3D7_ 0932200	PKNH_ 0730900	PbGEM- 286426
6.	*ttl(-)*	PBANKA_ 0901900	PF3D7_ 1147200	PKNH_ 0945000	PbGEM- 287138
7.	*tel DNA-bind protein(-)*	PBANKA_ 1205000	PF3D7_ 1006800	PKNH_ 0805600	PbGEM- 304250
8.	*bclp 1(-)*	PBANKA_ 0910100	PF3D7_ 1138600	PKNH_ 0936800	PbGEM- 288226
9.	*man-1-P-GT(-)*	PBANKA_ 1022300	PF3D7_ 1420900	PKNH_ 1337100	PbGEM- 037559
10.	*dgk(-)*	PBANKA_ 133460	PF3D7_ 1471400	PKNH_ 1209600	PbGEM- 265364
11.	*U2 snRNP(-)*	PBANKA_ 1039300	PF3D7_ 1402700	PKNH_ 1355400	PbGEM- 243939
12.	*conserved protein(-)*	PBANKA_ 0519500	No	No	PbGEM- 533923
13.	*dpap 1 (-)*	PBANKA_ 0931300	PF3D7_ 1116700	PKNH_ 0914400	PbGEM- 032467
14.	*hgprt(-)*	PBANKA_ 1210800	PF3D7_ 1012400	PKNH_ 0812200	PbGEM- 336339
15.	*protein kinase(-)*	PBANKA_ 0826900	PF3D7_ 0926100	PKNH_ 0724000	PbGEM- 259660
16.	*V-type(+) PPase(-)*	PBANKA_ 132050	PF3D7_ 1235200	PKNH_ 1454900	PbGEM- 253763
17.	*app(-)*	PBANKA_ 1318100	PF3D7_ 1454400	PKNH_ 1227700	n.a.
18.	*lap(-)*	PBANKA_ 1309900	PF3D7_ 1446200	PKNH_ 1236000	n.a.

Appendix Table S2. List of generated clonal *P. berghei* parasite lines. Gene IDs for *P. berghei* and homologues in *P. falciparum* and *P. knowlesi* as well as PlasmoGEM IDs used to generate the *Pb* lines are shown.

Appendix Table S3.

Gene ID	Primer	Sequence (5‘ → 3‘)
PBANKA_135800 (*trx 2*)	GT	TGGGGAATTCCATTGGCGGGA
	GT rev	CTTTGGTGACAGATACTAC
	QCR1	TCCGTGCTGCTTTGTTCCAA
	QCR2	AGCAGGATGTTTGTTTACCGGGGT
PBANKA_0615700 (*dynein heavy chain*)	GT	ACATTTCACGATGGCGCGGA
	GT rev	CTTTGGTGACAGATACTAC
	QCR1	TCCCCTTTGTGCCTTCAGAGCT
	QCR2	AGGAATCACCACGAAAGGGACA
PBANKA_0921700 (*atg 23*)	GT	ACGTGATGTGAATGCCTACA
	GT rev	TGATTAGCATAGTTAAATAAAAAAAGTTG
	QCR1	TCTTCACATTCTTCTTGCATGT
	QCR2	TTCACCCCCTACACCGATAT
PBANKA_0833000 (*profilin*)	GT	TGGCACACTTGGTTTGACAGAGGT
	GT rev	CATACTAGCCATTTTATGTG
	QCR1	TCATCGGGGGTTGCTACGCA
	QCR2	GCGTAAGGCTTCGTCCGTTT
PBANKA_0901900 (*ttl*)	GT	GGTTGTGCAAACCGAGACTT
	GT rev	TGATTAGCATAGTTAAATAAAAAAAGTTG
	QCR1	TTGGGTTGAGCCAGATTCGA
	QCR2	AGCATATTAGAGTCACAACCA
PBANKA_1205000 (*tel DNA-bind protein*)	GT	TGCAGTCGTATTTGTCACCACA
	GT rev	TGATTAGCATAGTTAAATAAAAAAAGTTG
	QCR1	ACGTCATTACATTTGTGGTAGCCA
	QCR2	ACCACTGAAGAAGGCGAAGA

PBANKA_0910100 (*bclp 1*)	GT	TGATGTGATGAGCAAATCCGA
	GT rev	TGATTAGCATAGTTAAATAAAAAAAGTTG
	QCR1	CGTCAATGTCAATCTCATCTTCATCGC
	QCR2	TCTGTTCCAGAAAATGGGGT
PBANKA_1022300 (*man-1-P-GT*)	GT	ACTTTGATGCCTGGCTCTCCT
	GT rev	CTTTGGTGACAGATACTAC
	QCR1	TGGGCAGATATTGGAAAACCTTCTGA
	QCR2	TCAATTCGAGCCCAGCTCCCT
PBANKA_1333460 (*dgk*)	GT	ACCAGAGGGTTGCCATTTGCACA
	GT rev	CTTTGGTGACAGATACTAC
	QCR1	AGCCCCAAATCAACACCTGAACGA
	QCR2	TGGGTGGAAGAAACCAATTGGGA
PBANKA_0519500 (*U2 snRNP*)	GT	ACCCTCGTTGCTCACATAACCGA
	GT rev	CATACTAGCCATTTTATGTG
	QCR1	CGGCATCTCCCTCAAACGACCG
	QCR2	ACGAAAACGCCCTTCACATCT
PBANKA_0519500 (*conserved protein*)	GT	TTGGAGGCGCTCTCATTGGT
	GT rev	GGCTATTCATACTAGCCATTTTATGTGTG
	QCR1	TGTGCTTTGACGGTTTAGCTCC
	QCR2	AAGAGACGCAAAAAGCACAC
PBANKA_0931300 (*dpap*)	GT	GCTGCTATCAATGCAGCACCACCA
	GT rev	CTTTGGTGACAGATACTAC
	QCR1	ACACGTGGTGCTGGTAAAGTGCT
	QCR2	TTAATGCAAGGGAGCCGACC

PBANKA_1210800 (*hgprt*)	GT	CCATCTTTCAAATGGCCCTCG
	GT rev	CATACTAGCCATTTTATGTG
	QCR1	AAAAGGGGCGTGCACAAATA
	QCR2	TGTTGCATGCGTTCCAGTTGT
PBANKA_0826900 (*protein kinase*)	GT	TGGGAATTGCGCATTTGGTGATCG
	GT rev	CATACTAGCCATTTTATGTG
	QCR1	TGTACCATCTTCCTCTGATT
	QCR2	GGAGGAATAGCCACATTACCTTCACG
PBANKA_132050 (*V-type H(+)-PPase*)	GT	AGTCATTTAGGAGCAGCGGA
	GT rev	CTTTGGTGACAGATACTAC
	QCR1	CGAGAGCGGGAGGACCAAAT
	QCR2	ATTGCCGTTCTAAAGCACTT
PF3D7_1454400 (*app*)	gRNA1 F	TATTTACCGTTGATGTTAACATGA
	gRNA1 R	AAACTCATGTTAACATCAACGGTA
	gRNA2 F	TATTATCCATCATGTTAACATCAA
	gRNA2 R	AAACTTGATGTTAACATGATGGAT
	HR1 For	GAGGTACCGAGCTCGAATTCAGAGGCAAAATTTAGTATCGG
	HR1 KO Rev	CACTATTAATTAAATTATTATTAATATATATTCTTAAATATATTGGAACCAC
	HR1 Ctrl gRNA1 Rev	GATTATTATCCATCATATTCACATCAACGGTATTATTATTACTCATC
	HR1 Ctrl gRNA1 Rev	CCATCATGTTAACATCAACTGTATTATTATTACTCATCATTTCGG
	HR2 KO For	TAAGAATATATATTAATAATAATTTAATTAATAGTGATGAACACAATTCTG
	HR2 Ctrl gRNA1 For	ATAATACCGTTGATGTGAATATGATGGATAATAATCCTGCTGC
	HR2 Ctrl gRNA2 For	TGATGAGTAATAATAATACAGTTGATGTTAACATGATGGATAATAATC
	HR2 Rev	CGAAAAGTGCCACCTGACGTCGGTGAATATTGATAATCATAACCTC
	Genotyping P1 For	GGTAAGAGGCAAAATTTAGTATCGG

	Genotyping P2 Rev	TAGTTCCATGTAAATATTGTCCTCCC
PF3D7_1446200 (*lap*)	gRNA1 F	TATTGCTGTTGGTTATGTAGGATG
	gRNA1 R	AAACCATCCTACATAACCAACAGC
	gRNA2 F	TATTGGAGGTTGTAATGTTGAAGA
	gRNA2 R	AAACTCTTCAACATTACAACCTCC
	HR1 gRNA1 For	GAGGTACCGAGCTCGAATTCTCCTAAAATCTACTATACACAGTGG
	HR1 KO gRNA1 Rev	ATCAGCAACTGATCTTATTAAACTGGCCCATTTTCTTTACCAG
	HR1 Ctrl gRNA1 Rev	CAACTGATCCACACCCGACGTACCCAACAGCAACTGAATTTTTATTA TC
	HR2 KO gRNA1 For	AAAATGGGCCAGTTTAATAAGATCAGTTGCTGATTTAAGTGAAGC
	HR2 Ctrl gRNA1 For	TTGCTGTTGGGTACGTCGGGTGTGGATCAGTTGCTGATTTAAGTG
	HR2 gRNA1 Rev	CGAAAAGTGCCACCTGACGTCGCCACAATAGATGAAGCTTTAACAC
	HR1 gRNA2 For	GAGGTACCGAGCTCGAATTCGCCTTTGTGCATGGTTAATAATAATG
	HR1 KO gRNA2 Rev	ATTTTCTTTACCAGTTATTATGGATCTAAGGAAACAACTTGTGG
	HR1 Ctrl gRNA2 Rev	TTAATCCTTCCTCGACGTTGCAACCTCCTTTTATATCATAAACCTGAA C
	HR2 KO gRNA2 For	TTTCCTTAGATCCATAATAACTGGTAAAGAAAATGGGCCAG
	HR2 Ctrl gRNA2 For	AAGGAGGTTGCAACGTCGAGGAAGGATTAACTATTTTCTTAGTTAAT AATCCTGG
	HR2 gRNA2 Rev	CGAAAAGTGCCACCTGACGTCCCCACAGATAAATAGGCTCCC
	Genotyping P1 For	ATCCTAAAATCTACTATACACAGTGG
	Genotyping P2 Rev	CAATATCAATATGTGCCCAAGC
PF3D7_0805700 (*fikk8*)	gRNA F	TATTATGGATCCTAAAGATTGTTC
	gRNA R	AAACGAACAATCTTTAGGATCCAT
	HR1 For	GAGGTACCGAGCTCGAATTCAAGTAATGTTGATATGTGTGAGGG
	HR1 KO Rev	ATCTTCATCCTGTTCCTTATTATTAATCCTTATTTGTATCTTCCTTCAT C
	HR1 Ctrl Rev	TCATTAAAATTTATTCCGCTACAATCTTTAGGATCCATAGAAATATTT C

	HR2 KO For	AGATACAAATAAGGATTAATAATAAGGAACAGGATGAAGATGAAGG
	HR2 Ctrl For	TGGATCCTAAAGATTGTAGCGGAATAAATTTTAATGAAATG
	HR2 Rev	CGAAAAGTGCCACCTGACGTCCCTTAACATCCACTTGTTCATAAC
	Genotyping P1 For	ATGAATGATGCTGAAGATGTGTC
	Genotyping P2 Rev	GCATATCATTTTGACCTGCTCC
PF3D7_0725200 (*mago nashi*)	gRNA F	TATTACACTGTTTAACATCTGATA
	gRNA R	AAACTATCAGATGTTAAACAGTGT
	HR1 For	GAGGTACCGAGCTCGAATTCCCTTTAGATATTAGTAATTGAAATAAGAGC
	HR1 KO Rev	TCTGGGTCATTATTATTACACTTTACATATTTCAGATTCTTCAATTATACG
	HR1 Ctrl Rev	CTTGTTTTCCAATCTTATCAGGCATAGGCCATTCTTTATCAC
	HR2 KO For	TATGTAAAGTGTAATAATAATGACCCAGAAGGTTTACGTG
	HR2 Ctrl For	CTACTTCTAAAATTGGATCGTTATCAGATGTTAAACAGTGTAGTG
	HR2 Rev	CGAAAAGTGCCACCTGACGTCTTAATAAGGTACATTTACGTTCTTGG
	Genotyping P1 For	AATGTCTAAGAAAGATAAATTTTCCTTTAG
	Genotyping P2 Rev	TTAATAAGGTACATTTACGTTCTTGG
PF3D7_1148800 (*hyp11*)	gRNA F	TATTAAGGTATAGAAGAATAGTAG
	gRNA R	AAACCTACTATTCTTCTATACCTT
	HR1 For	GAGGTACCGAGCTCGAATTCTGTGGTTGCTTGATCTGGAC
	HR1 KO Rev	TTGCATATTTACCTTTATTATGTTTCATTGAAAGGGTAATATAGTTG
	HR1 Ctrl Rev	CATGTTGTTCTTCGGCGACGATTCTTCTATACCTTGAGCATGTTTC
	HR2 KO For	CTTTCAATGAAACATAATAAAGGTAAATATGCAACAAGATGGTG
	HR2 Ctrl For	GGTATAGAAGAATCGTCGCCGAAGAACAACATGAAATTGAATTAGACG
	HR2 Rev	CGAAAAGTGCCACCTGACGTCCCATTAAGGTAATAATTTACTTCCTCCATC
	Genotyping P1 For	TGTGGTTGCTTGATCTGGAC
	Genotyping P2 Rev	TCTTTCTAAATAATGTTCCGGTTCATC

PF3D7_0828500 (*eif-2b*)	gRNA F	TATTTTACTGGAGTCGCTAAAATG
	gRNA R	AAACCATTTTAGCGACTCCAGTAA
	HR1 For	GAGGTACCGAGCTCGAATTCCCTATTCAACCTTTGCCAAGAG
	HR1 KO Rev	ATCTTCTGTGGTGCTTATTAGCAACTGTTCAATGTCTTTGC
	HR1 Ctrl Rev	AATTACTGGAGTCTGAGAAGTGAGGACATTCATTTAATAAGCAACTG
	HR2 KO For	CATTGAACAGTTGCTAATAAGCACCACAGAAGATCACAATG
	HR2 Ctrl For	ATGAATGTCCTCACTTCTCAGACTCCAGTAATTTGGAATACCG
	HR2 Rev	CGAAAAGTGCCACCTGACGTCAAATGATACTAGTACCTACATGCCC
	Genotyping P1 For	CCTATTCAACCTTTGCCAAGAG
	Genotyping P2 Rev	TTCGAATGATGATGATAATGATGC
PF3D7_1327600 (*nmnat*)	gRNA1 F	TATTATTTCGTGAGCATATGTAAT
	gRNA1 R	AAACATTACATATGCTCACGAAAT
	gRNA2 F	TATTTCTGTGATGAAATTCTGTTA
	gRNA2 R	AAACTAACAGAATTTCATCACAGA
	HR1 gRNA1 For	GAGGTACCGAGCTCGAATTCGACCCTTTATCCTTCCTATAACCC
	HR1 KO gRNA1 Rev	TATGTCTGTGATGATTATTACTTATGCATTTTAAAGGGACCAC
	HR1 Ctrl gRNA1 Rev	CCATTTCGTGAGCGTACGTGATTGGATCAAAGGACCCTCC
	HR2 KO gRNA1 For	TTAAAATGCATAAGTAATAATCATCACAGACATAACATGTTTACC
	HR2 Ctrl gRNA1 For	CCTTTGATCCAATCACGTACGCTCACGAAATGGTTCTTG
	HR2 gRNA1 Rev	CGAAAAGTGCCACCTGACGTCTGCATAATACAAGTACGAGAAGGG
	HR1 gRNA2 For	GAGGTACCGAGCTCGAATTCGACCCTTTATCCTTCCTATAACCC
	HR1 KO gRNA2 Rev	TATGAGATTCAAGATTATTATGTAATTGGATCAAAGGACCCTC
	HR1 Ctrl gRNA2 Rev	TGATGAAATTCTGTCAATGATTTATCATTTCTACATCTACATATAACTA CCC
	HR2 KO gRNA2 For	TTGATCCAATTACATAATAATCTTGAATCTCATAGTGAAATGACTCC

	HR2 Ctrl gRNA2 For	GTAGAAATGATAAATCATTGACAGAATTTCATCACAGACATAACATG
	HR2 gRNA2 Rev	CGAAAAGTGCCACCTGACGTCTGCATAATACAAGTACGAGAAGGG
	Genotyping P1 For	GACCCTTTATCCTTCCTATAACCC
	Genotyping P2 Rev	TGTATATATGTGCAATGGGTTAATGTTC
PF3D7_1345100 (*trx2*)	gRNA F	TATTAAAGCGCATATACTTATTAA
	gRNA R	AAACTTAATAAGTATATGCGCTTT
	HR1 For	GAGGTACCGAGCTCGAATTCGTGTACGATGTTACATGTACAAAAG
	HR1 KO Rev	CATTGCTTGAGACATTATTAACAAGCTTGACACCTACACAC
	HR1 Ctrl Rev	TTGTCTAGGTCAACTTTGAGTAAGTATATGCGCTTTCCGTAATATTTTTG
	HR2 KO For	GGTGTCAAGCTTGTTAATAATGTCTCAAGCAATGATTTGATAGC
	HR2 Ctrl For	AGCGCATATACTTACTCAAAGTTGACCTAGACAAAAATGAATCAC
	HR2 Rev	CGAAAAGTGCCACCTGACGTCATATAGACCTGTTGCATTTGCC
	Genotyping P1 For	CTATAGCATATATAAAGATTATATATCGAAACAAC
	Genotyping P2 Rev	ATATAGACCTGTTGCATTTGCC

Appendix Table S3. Primers used in this study.

Appendix Table S4.

	PlasmoGEM ID	Design warning	Coding sequence remaining in genome (%)
trx2(-)	PbGEM-317410	yes	54
dynein heavy chain(-)	PbGEM-330283	yes	25
atg 23(-)	PbGEM-332739	yes	15
profilin(-)	PbGEM-286426	no	n.a.
ttl(-)	PbGEM-287138	yes	20
tel DNA-bind protein(-)	PbGEM-304250	no	n.a.
bclp 1(-)	PbGEM-288226	no	n.a.
man-1-P GT(-)	PbGEM-037559	yes	48
dgk (-)	PbGEM-265364	yes	56
U2 snRNP(-)	PbGEM-243939	yes	33
conserved protein(-)	PbGEM-533923	no	n.a.
dpap 1(-)	PbGEM-032467	no	n.a.
hgprt(-)	PbGEM-336339	yes	<1
protein kinase(-)	PbGEM-259660	yes	21
V-type(+) PPase(-)	PbGEM-253763	no	n.a.

Appendix Table S4. Overview of design warnings for all generated mutants.

Appendix Table S5.

	In vivo multiplication rate				Oocysts/ infected midgut (mean)	Infected midguts/ midguts analyzed [%]	salivary gland sporozoites / mosquito
	Plasmo GEM	95% CI	clone	95% CI			
PbA	1	n.a.	1±0.11	0.79-1.21	58	88	10,400–27,500
trx 2(-)	0.75	0.56 – 0.95	1.05±0.23	0.59-1.5	239	52	9,200 – 13,000
plasmepsin IV(-)	0.67	0.66 – 0.69	0.58#	n.a.	33	57	0
dynein heavy chain(-)	0.6	0.22 – 0.97	0.8±0.05	0.72-0.91	55	68	11,000 – 23,000
atg 23(-)	0.58	0.49 – 0.68	0.7±0.04	0.61-0.77	47	82	14,000 – 15,000
profilin(-)	0.57	0.45 – 0.69	0.6	n.a.	15	35	0
ttl(-)	0.51	-0.23 – 1.26	0.7±0.04	0.65-0.82	12	65	200 – 6,000
tel DNA-bind protein(-)	0.44	0.33 – 0.56	0.8±0.07	0.67-0.94	0	0	0
bclp 1(-)	0.42	0.35 – 0.49	0.6±0.05	0.46-0.67	0	0	0
man-1-P GT(-)	0.39	0.27 – 0.52	0.8±0.04	0.85-0.89	0	0	0
dgk(-)	0.37	0.25 – 0.48	0.7±0.05	0.56-0.75	39	72	0 – 900
U2 snRNP(-)	0.39	0.31 – 0.48	0.5±0.02	0.42-0.48	0	0	0
conserved protein(-)	0.35	-0.12 – 0.82	0.9±0.18	0.58-1.27	119	76	5,500 – 15,000
dpap 1(-)	0.33	-0.12 – 0.78	0.9±0.07	0.72-0.98	82	76	850 – 7,000
hgprt(-)	0.29	0.12 – 0.45	0.4±0.03	0.38-0.48	0	0	0
protein kinase(-)	0.29	0 – 0.58	0.8±0.07	0.63-0.91	24	70	5,500 – 11,000
V-type(+) PPase(-)	0.27	0.22 – 0.32	0.5	n.a.	42	72	550 – 5,100
app(-)	0.21	0.07 – 0.35	0.46*	n.a.	25	65	1,600 – 11,000
lap(-)	0.25	0.14 – 0.36	0.33*	n.a.	26	52	1,500 – 6,000

Appendix Table S5. Infection overview across life cycle. For *in vivo* multiplication rates, growth rates from limiting dilutions were normalized to intra experimentally obtained wild type multiplication rates (Appendix Fig. S3). # indicates multiplication rate as reported in (Spaccapelo *et al*, 2010) normalized to reported wild type multiplication rate. * indicate multiplication rates as reported in (Lin *et al*, 2015)normalized to reported wild type multiplication rate. Mean sporozoite numbers from at least two independent mosquito infections. CI, confidence interval; n.d., not determined.

Appendix Table S6.

	100 iRBC SWISS			100 iRBC C57BL/6		
	Mice clearing/ mice infected (prepatency d)	Peak parasitemia cleared [%] (mean d)	Blood stage cleared (mean d)	Mice clearing/ mice infected (prepatency d)	Peak parasitemia cleared [%] (mean d)	Blood stage cleared (mean d)
thioredoxin 2(-)	0/4 (7)	n.a.	n.a.	n.d.	n.a.	n.a.
plasmepsin IV(-)	1/3 (6)	21 (17)	25	n.d.	n.a.	n.a.
dynein heavy chain(-)	0/4 (4)	n.a.	n.a.	0/4	n.a.	n.a.
atg 23(-)	2/4 (6)	32 (19)	23	n.d.	n.a.	n.a.
profilin(-)	0/4 (7)	n.a.	n.a.	3/4 (6)	10 (15)	23
ttl(-)	0/4 (5)	n.a.	n.a.	n.d.	n.a.	n.a.
tel DNA-bind protein(-)	0/3 (5)	n.a.	n.a.	n.d.	n.a.	n.a.
bclp 1(-)	3/4 (6)	42 (19)	37	n.d.	n.a.	n.a.
man-1-P-GT(-)	0/3 (6)	n.a.	n.a.	3/8 (7)	41 (25)	34
U2 snRNP(-)	6/6 (9)	15 (19)	26	8/8 (8)	9 (19)	25
dgk(-)	3/4 (6)	22 (18)	27	4/4 (5)	17 (19)	28
conserved protein(-)	0/4 (5)	n.a.	n.a.	n.d.	n.a.	n.a.
dpap1(-)	0/4 (6)	n.a.	n.a.	n.d.	n.a.	n.a.
hgprt(-)	4/4 (8)	8 (17)	23	4/4 (9)	1 (15)	17
protein kinase(-)	0/4 (6)	n.a.	n.a.	0/4 (6)	n.a.	n.a.
V-type(+) PPase(-)	1/4 (7)	21 (13)	21	n.d.	n.a.	n.a.

	Natural transmission C57BL/6			1,000 salivary gland sporozoites i.v. C57BL/6			10,000 salivary gland sporozoites i.v. SWISS		
	mice clearing/ mice infected (prepatency d)	Peak parasitemia cleared [%] (mean d)	Blood stage cleared (mean d)	mice clearing/ mice infected (prepatency d)	Peak parasitemia cleared [%] (mean d)	Blood stage cleared (mean d)	mice clearing/ mice infected (prepatency d)	Peak parasitemia cleared [%] (mean d)	Blood stage cleared (mean d)
dynein heavy chain(-)	n.d.	n.a.	n.a.	0/4 (4)	n.a.	n.a.	0/4 (6)	n.a.	n.a.
atg 23(-)	n.d.	n.a.	n.a.	0/4 (5)	n.a.	n.a.	0/4 (5)	n.a.	n.a.
ttl(-)	n.d.	n.a.	n.a.	0/4 (4)	n.a.	n.a.	0/4 (6)	n.a.	n.a.
conserved protein(-)	0/3 (4)	n.a.	n.a.	0/3 (4)	n.a.	n.a.	n.d.	n.a.	n.a.
protein kinase(-)	0/6 (5)	n.a.	n.a.	0/3 (5)	n.a.	n.a.	n.d.	n.a.	n.a.

Appendix Table S6. Overview of infections with blood stage or salivary gland derived sporozoites from indicated mutants in SWISS and C57BL/6 mice. Natural transmission refers to infection by the bite of 10 infected mosquitoes. Abbreviations: n.a. not applicable, n.d. not determined.

Appendix Table S7.

Immunized through			Animals infected/ animals challenged	Average prepatency [d]	Average peak parasitemia [%]	Animals dying/ animals challenged
Sporozoites i.v.	*app(-)*	d90	6/8	10	0.9	0/8
		d180	3/8	10	0.5	0/8
		d 360[1]	4/4	8	2.3	0/4
	lap(-)	d90	2/8	10	0.7	0/8
		d180	6/8	8	0.8	0/8
		d 360[1]	1/4	6	0.7	0/4
Natural transmission	*app(-)*	d90	2/7	12	0.4	0/7
		d180	2/4	10	0.3	0/4
	lap(-)	d90	3/8	10	0.3	0/8
		d180	4/4	11	0.7	0/4

[1] mice already challenged at d180 were rechallenged at d360

Appendix Table S7. Summary of all challenged mice and the route of immunization from experiments reported in Fig. 3.

Appendix Table S8

Challenge with 1,000 spz			Animals infected/ animals challenged	Average prepatency [d]	Average peak parasitemia [%]	Animals dying/ animals challenged
P. berghei	naive control	d90	4/4	4.5	6.1	4/4
	NK65 + CQ	d90	4/4	4.8	1.8	0/4

Appendix Table S8. Overview of challenge of NK65 and chloroquine immunized mice 90 days post immunization.

Appendix Table S9.

Gene ID	Gene	Description	rGR	MIS	MFI	# Transf. (gRNAs)	# Transf. Succ.	eGR
PF3D7_ 0725200	mago nashi	mago nashi protein homologue, putative	0.16	1	-2.2	2 (2)	1	0.98 ± 0.09
PF3D7_ 1454400	app	Amino-peptidase P	0.21	0.15	-3.3	3 (2)	0	-
PF3D7_ 1446200	lapp	M17 leucylamino-peptidase	0.25	0.12	-3.0	2 (2)	0	-
PF3D7_ 0805700	fikk8	serine/ threonine protein kinase, FIKK family	0.3	1	-2.7	2 (2)	1	0.69 ± 0.02
PF3D7_ 1327600	nmnat	Nicotinamide mono Nucleotide adenylyl transferase	0.41	0.75	-2.9	1 (2)	2	1.10 ± 0.11<br>1.42 ± 0.02
PF3D7_ 0828500	eif-2b	TIF eIF-2B delta subunit	0.51	0.14	-2.0	1 (2)	1	0.95 ± 0.01
PF3D7_ 1345100	trx2	Thioredoxin 2	0.75	0.12	-3.2	1 (1)	1	0.8 ± 0.04
PF3D7_ 1148800	hyp11	Plasmodium exported protein	no data	1	-2.5	1 (1)	1	0.94 ± 0.02

Appendix Table S9. Growth characterisation of *P. falciparum* knockout lines. Predicted relative (rGR; (Zhang *et al*, 2018)) and experimental (eGR; this study) growth rates of parasite lines, along with mutagenesis index score (MIS) and mutagenesis fitness index (MFI) reported previously (Zhang *et al*, 2018). transf., transfection; gRNA, guide RNA; succ., success.

Appendix Table S10.

Figure			Replicates[1]	P value	Statistical test
1	D	*app(-)*	3	0.0047	Shapiro-wilk normality test followed by a Kruskal-Wallis test
		lap(-)	3	0.0319	
5		IFNγ	2	0.0366	Unpaired t-test
		TNA-α	2	0.0016	
		IL-12p70	2	0.044	
		IL-1 β	2	0.0046	
		IL-17A	2	0.0076	
		MCP-1	2	0.0027	
6	B	*mago nashi*	3	0.754924	Unpaired t-test with Holm-Sídák`s correction
		fikk8	3	0.002409	
		nmnat	2x3	0.029129	
		eif-2b	3	0.163771	
		trx2	3	0.028974	
		hyp11	3	0.495731	
S3	A	*PbA*	7		Sídák`s multiple comparison test
		trx2(-)	3	0.9961	
		plasmepsinIV(-)	1	0.0352	
		dynein heavy chain(-)	5	0.0257	
		atg 23(-)	5	<0.0001	
		profilin(-)	1	0.0052	
		ttl(-)	3	0.0037	
		bclp 1(-)	2	<0.0001	
		tel DNA-bind protein (-)	6	0.01	
	B	*man-1-P GT(-)*	3	0.0927	Sídák`s multiple comparison test
		U2 snRNP(-)	3	<0.0001	
		dgk(-)	4	<0.0001	
		conserved protein(-)	4	0.8638	
		dpap1(-)	3	0.2222	
		hgprt(-)	3	<0.0001	
		protein kinase(-)	3	0.013	
		V-type (+) PPase(-)	1	0.0010	
		lap(-)	1	0.0001	
		app(-)	1	<0.0001	
S4	A	*trx2(-)*	2	<0.0001	Two-tailored Mann-Whitney test
		pm IV(-)	2	0.0738	
		dynein heavy chain(-)	3	0.5198	

	atg 23(-)	3	0.5285	
	ttl(-)	4	<0.0001	
	dgk(-)	2	0.1215	
	conserved protein(-)	2	0.0006	
	dpap1(-)	2	0.0005	
	protein kinase(-)	2	0.0004	
	V-type(+) PPase(-)	2	0.2816	
D	*trx2(-)*	2	<0.0001	Two-tailored Mann-Whitney test
	dynein heavy chain(-)	2	0.0657	
	atg 23(-)	2	<0.0001	
	ttl(-)	2	<0.0001	
	conserved protein(-)	2	<0.0001	
	dpap 1(-)	2	<0.0001	
	protein kinase(-)	2	<0.0001	
	V-type (+) PPase(-)	2	<0.0001	

[1]biological replicates with the exceptions of Figure 5, which are technical replicates

Appendix Table S10. Summary of all p-values reported.
