# Supplementary figures and images for: Experimental vaccination by single dose sporozoite injection of blood-stage attenuated malaria parasites
Source: EMBO Mol Med. 2024 Aug 5;16(9):2060–79. doi: 10.1038/s44321-024-00101-6 (PMC11392930; doi:10.1038/s44321-024-00101-6)

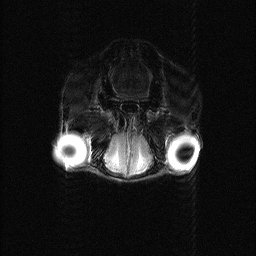

Supplement: Supplementary file 6 — Source data Fig. 2 [file 44321_2024_101_MOESM6_ESM.zip › Figure 2/Figure 2J/MRIm19 WT d7.jpg]

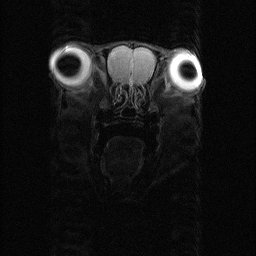

Supplement: Supplementary file 6 — Source data Fig. 2 [file 44321_2024_101_MOESM6_ESM.zip › Figure 2/Figure 2J/MRIm12 app d14.jpg]

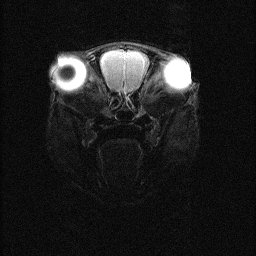

Supplement: Supplementary file 6 — Source data Fig. 2 [file 44321_2024_101_MOESM6_ESM.zip › Figure 2/Figure 2J/MRIm18 app d7.jpg]

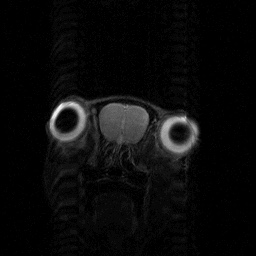

Supplement: Supplementary file 6 — Source data Fig. 2 [file 44321_2024_101_MOESM6_ESM.zip › Figure 2/Figure 2J/MRIm13 lap 819_1 d14.jpg]

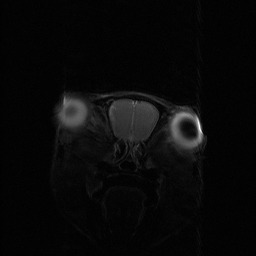

Supplement: Supplementary file 6 — Source data Fig. 2 [file 44321_2024_101_MOESM6_ESM.zip › Figure 2/Figure 2J/MRIm12 lap 819_1 d6.jpeg]
