# Supplementary material for: Experimental vaccination by single dose sporozoite injection of blood-stage attenuated malaria parasites
Source: EMBO Mol Med. 2024 Aug 5;16(9):2060–79. doi: 10.1038/s44321-024-00101-6 (PMC11392930; doi:10.1038/s44321-024-00101-6)
Supplement: Supplementary file 12 — Expanded View Figures [file 44321_2024_101_MOESM12_ESM.pdf]

Expanded View Figure

Figure EV1. Prepatency, parasitemia curves and survival of C57BL/6 mice infected with 1000 intraerythrocytic parasites or 10,000 sporozoites of PbA or *lap(-)*.

(**A**) Percentage of blood-stage negative C57BL/6 mice infected with either 1000 PbA or *lap(-)* iRBC ($n = 4$ each) or 10,000 PbA or *lap(-)* sporozoites ($n = 4$ each). All C57BL/6 became infected between d4 and d7. (**B**) Parasitemia of mice ($n = 4$) infected in (**A**). Parasitemia is shown as mean parasitemia. Left panel: 2 of 4 mice infected with 1000 PbA iRBC died of ECM and 2 were not developing ECM and were dying on day 18 post-infection. All mice infected with 1000 *lap(-)* iRBC cleared the infection. Right panel: Mean parasitemia of all mice infected with 10,000 PbA or *lap(-)*sporozoites. Mean parasitemia values ± standard deviations from all infected mice are shown. (**C**) Rapid murine coma and behavioral score (RMCBS) of all mice ($n = 4$) infected in (**A**). Two of four mice infected with 1000 PbA iRBC (left panel) or 10,000 PbA sporozoites (right panel) were developing ECM symptoms starting on day 6 post-infection. All other mice were not showing a decrease of RMCBS. Mean RMCBS values ± standard deviations from all infected mice are shown. (**D**) Body weight of all mice ($n = 4$) infected in (**A**). Mice infected with 1000 PbA iRBC (left panel) or 10,000 PbA sporozoites (right panel) and developing ECM also displayed a loss of body weight until death. Two mice infected with 1000 PbA iRBC (left panel) but not developing ECM initially lost weight but then recovered followed by another loss in weight until dying on day 18 post-infection. In contrast all mice infected with either 1000 *lap(-)* iRBC (left panel) or 10,000 *lap(−)* sporozoites (right panel) recovered from temporary body weight loss. Mean body weight ± standard deviations from all infected mice is shown. (**E**) Survival of mice ($n = 4$) infected in (**A**). Mice infected with 1000 PbA iRBC died either on day 9 post-infection due to ECM or on day 18 (left panel) due to anemia. Mice infected with 10,000 PbA sporozoites (right panel) died between day 8 and 9 post-infection due to ECM. All mice infected with either 1000 *lap(−)* iRBC (left panel) or 10,000 *lap(-)* sporozoites (right panel) cleared the infection and survived. (**F**) Summary of PbA wild-type and *lap(−)* infections of C57BL/6 mice ($n = 4$) infected with 1000 iRBCs or 10,000 sporozoites. Numbers for peak parasitemia and days are averages from the indicated number of investigated mice. Source data are available online for this figure

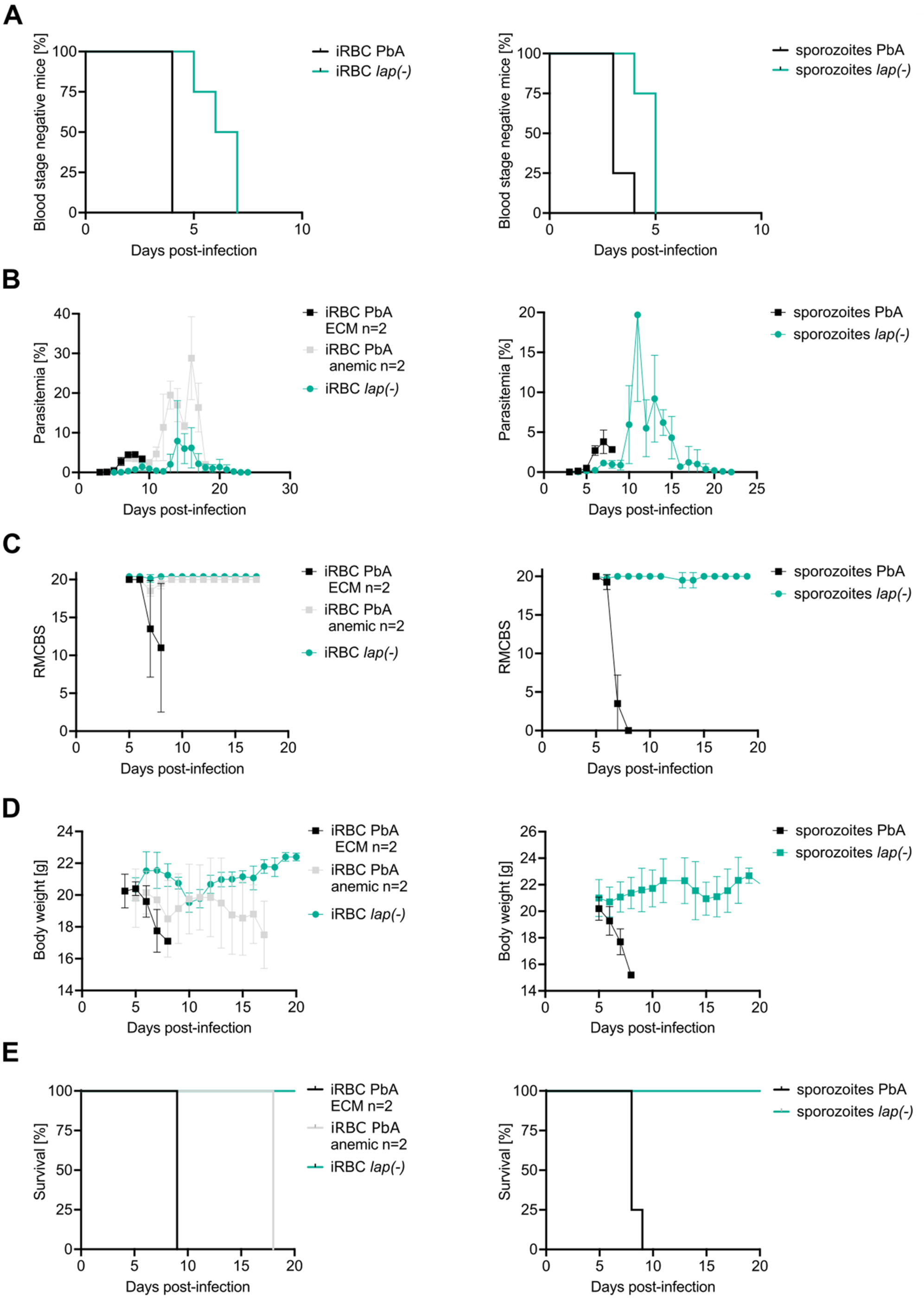

F

	1,000 iRBC		10,000 sporozoites	
	Peak parasitemia cleared [%] (mean d)	Blood stage cleared (mean d)	Peak parasitemia cleared [%] (mean d)	Blood stage cleared (mean d)
PbA	n.a.	0/4	n.a.	0/4
lap(-)	11.1 (15)	4/4 (23)	20.7 (12)	4/4 (20)

 © The Author(s)
